# The effectiveness of thunder fire moxibustion for treating allergic rhinitis: A protocol for systematic review and meta analysis

**DOI:** 10.1097/MD.0000000000020711

**Published:** 2020-07-24

**Authors:** Jun Xiong, Ting Yuan, Qiaotong Huang, Xue Wang, Jun Yang, Yunfeng Jiang, Xiaohong Zhou, Kai Liao, Lingling Xu

**Affiliations:** a Department of Acupuncture and Moxibustion, The Affiliated Hospital with Jiangxi University of TCM; b School of Acupuncture, Moxibustion and Tuina of Jiangxi University of Traditional Chinese Medicine, Nanchang; c The First Affiliated Hospital of Guangxi University of Traditional Chinese Medicine, Nanning, China.

**Keywords:** allergic rhinitis, protocol, systematic review and meta analysis, thunder fire moxibustion

## Abstract

**Background:**

Allergic rhinitis (AR) is a chronic disease, resulting in severe syndromes such as sneezing, itching, nasal blockage, and rhinorrhea. The major medications treating AR cause side effects, while thunder fire moxibustion (TFM) is known as a safe and effective treatment for AR. Thus, this systematic review and meta analysis aims to systematically evaluate the effectiveness and safety of TFM in the treatment of AR.

**Methods:**

Nine databases, including Medline, PubMed, Web of Science, Embase, the Cochrane Library, China National Knowledge Infrastructure Database (CNKI), WanFang Database (WF), Chinese Scientific Journal Database (VIP), and Chinese Biomedical Literature Database (CBM) from inception to July 2020 will be searched. In addition, the grey literature and the references of all included literature will be retrieved manually. Reviewers will identify studies, extract data and assess the quality independently. The outcomes of interest include: total nasal symptom score, total non-nasal symptom score, rhinitis quality of life questionnaire, visual analog scale, Laboratory indicators: serum immunoglobulin E, immunoglobulin A, or immunoglobulin G level and adverse events. Randomized clinical trials will be collected, methodological quality will be evaluated using the Cochrane risk-of-bias assessment tool, and the level of evidence will be rated using the Grading of Recommendations, Assessment, Development and Evaluation (GRADE) approach. Meta-analysis will be performed using RevMan5.3.0 software.

**Results:**

Because the review is ongoing, no results can be reported.

**Conclusions:**

The findings from this review will provide reliable evidence for effectiveness and safety of TFM for AR.

**Ethics and dissemination:**

Ethical approval requirements are not necessary for this review. This systematic review and meta analysis will be disseminated online and on paper to help guide the clinical practice better.

**PROSPERO Registration number:**

CRD42019141113

## 1 Introduction

Allergic rhinitis (AR) is a prevalent noninfectious inflammatory disease, which caused by allergic individuals exposed to allergens and mainly characterized by allergic inflammation of nasal mucosa.^[1]^ In AR, the degranulation of immunoglobulin E (IgE)-mediated mast cell and the release of mediators cause a rapid response, resulting in sneezing, itchy palate, nasal blockage, runny nose, and nasal hemorrhoids, which might be related to eye symptoms, including itchy eyes, red eyes, watering, and burning sensation. An inflammatory reaction with eosinophilic infiltration may occur in later stages.^[2–4]^ AR is common and affects the quality of life^[5]^ by interfering with normal work and study of an individual,^[6,7]^ thereby to huge NHS (National Health Service) costs.^[8]^ AR is often accompanied by asthma attacks. It is clinically diagnosed based on detailed medical history, specialized examination, and laboratory tests to assess serum IgE, immunoglobulin A, or immunoglobulin G levels. The widespread of AR has attracted global attention. In recent years, the incidence of AR has been on a sharp rise, currently affecting about 10% to 20% of the population worldwide.^[9]^ Epidemiological surveys show that the prevalence of AR in American adults ranges from 10% to 30%.^[10,11]^ Based on the impact on asthma (ARIA), the prevalence of AR is 40% to 50% in the general population of Europe and the USA^[10]^ and 4% to 38% in that of mainland China.^[12]^ Furthermore, AR is more likely to occur in individuals with atopy and family history of rhinitis, as well as in first-born children and immigrants.^[13–15]^ Although the disease is common in children, it accounts for about one-third of the adult rhinitis cases. Nowadays, the treatment methods for AR are mainly western medicines.^[16]^ Among them, commonly used western medicines include corticosteroids, antihistamines, and mast cell membrane stabilizers, etc. Although these medicines can temporarily alleviate nasal symptoms, they cannot completely cure AR. Moreover, the medicines have significant side effects, such as drowsiness, dry mouth, and cardiac toxicity caused by antihistamines, etc. Therefore, the medicines should not be taken for a long time.^[17]^ However, nonpharmacological therapy, including complementary and alternative medicines, for instance, thunder fire moxibustion (TFM), also has a good clinical effect.^[18]^

TFM is also known as the thunder-fire needle. It is made of moxa sticks with traditional Chinese medicine powder and moxa wool. It is like a big firecracker. After lighting, 10 layers of cotton paper were placed on the acupuncture points to press and warm moxibustion. It acts on moxibustion acupoints by burning, generating big fire power, infrared thermal radiation and medicinal effects. The treatment characteristics of TFM are as follows: First, according to the principle of TCM syndrome differentiation and treatment, a variety of drugs are used to make different kinds of TFM columns. Second, the use of a variety of therapeutic techniques, such as finches and array methods. Third, when burnt, TFM produces a powerful potency, with medicines that are rapidly adsorption on the human body surface, forming high-consistency medicine in areas around the skin and penetrating the acupuncture points and through the human body meridian transmission to improve the treatment effect.^[19]^ With the help of thermal radiation, TFM can improve circulation by penetrating deeply into the tissue through heat.^[20]^

Although several clinical trials have been conducted on TFM for treating AR, no systematic review and meta-analysis of TFM or TFM combined with western medicine for treating AR are yet reported. Intriguingly, many high-quality clinical trials have reported that western medicine has serious adverse reactions and long-term use is prone to drug resistance, but TFM has fewer adverse reactions and higher safety. Hence, the goal of this systematic review and meta-analysis is to assess the quality of these randomized controlled trials (RCTs) so as to evaluate the effectiveness and safety of TFM for treating AR and guide clinicians better.

## 2 Methods

### 2.1 Study registration

This systematic review and meta analysis will be performed according to the guidelines of the Cochrane Handbook for Systematic Reviews and Meta-Analysis Protocol (PRISMA-P).^[21]^ The protocol was beforehand registered in PROSPERO 2019 CRD42019141113. And it could be found from http://www.crd.york.ac.uk/PROSPERO/display_record.php?ID=CRD42019141113.

Any changes in the standard protocol will be described further.

### 2.2 Inclusion criteria

#### 2.2.1 Types of studies

Only relevant RCTs or quasi-RCTs published on TFM for treating AR will be included.

#### 2.2.2 Types of participants

Participants follow stringent diagnostic criteria according to ARIA,^[22]^ and those diagnosed with AR are eligible for inclusion in this review. No limitation will be set on the patients’ age, gender, occupation, ethnic group, disease duration, syndrome type, source of cases, or disease severity. The studies in which AR is combined with other allergic or basic diseases will be excluded.

#### 2.2.3 Types of interventions

TFM as a single intervention or primary part of a combination therapy with other positive treatments (eg, western medicine, conventional therapy etc) will be included. Interestingly, no restrictions are placed on the number of acupoints, the method of moxibustion, duration, and frequency. However, the clinical trials of TFM as an adjunctive therapy will be excluded.

#### 2.2.4 Types of comparator(s)/control

The comparators will include positive treatments (eg, western medicine and conventional therapy etc), no therapy, placebo or sham TFM. The specific forms of choice include the following:

(1) TFM VS positive treatments;
(2) TFM+ positive treatments VS positive treatments;
(3) TFM VS no therapy;
(4) TFM VS placebo;
(5) TFM VS sham TFM.

#### 2.2.5 Types of outcome measures

##### 2.2.5.1 Primary outcomes

The total nasal symptom score^[23]^ will be used to evaluate the score by 5 degree of 4 nasal symptoms (rhinorrhea, nasal itching, nasal obstruction, and sneezing).

##### 2.2.5.2 Secondary outcomes

(1) Total nonnasal symptom score^[23]^;
(2) Rhinitis quality of life questionnaire^[24]^;
(3) Visual analog scale: Patients grade their symptoms by using a swimming scale about 10 cm long, with 10 scales on one side and 0 and 10 on both ends, 0 indicating “no symptoms” and 10 indicating “‘the most severe symptoms;”
(4) Laboratory indicators: serum IgE, immunoglobulin A, or IgG level;
(5) Adverse events or side effects.

### 2.3 Exclusion criteria

Duplicate detection and republished literature; TFM plus other nonpharmacological treatment; expert experience or case reports; theoretical research or experimental research; unclear diagnostic criteria and efficacy evaluation criteria; conference papers and incomplete data of the results.

### 2.4 Search methods

#### 2.4.1 Electronic searches

Five English databases, including Medline, PubMed, Web of Science, Embase, and the Cochrane Library and four Chinese databases, including CNKI, WanFang, VIP, and CBM from the inception to July 2020 will be searched without any language restriction, but involving only the human subjects. The main keywords include “thunder fire moxibustion,” “allergic rhinitis,” and “RCT.” In addition, the searches will be rerun prior to the final analysis that followed the PRISMA protocol.

#### 2.4.2 Searching other resources

Reference lists of this study will be retrieved manually. We will also search relevant conference summaries for qualified trials. Moreover, the grey literature and the ongoing and recently completed research will be searched at clinicaltrials.gov.

#### 2.4.3 Searching strategy

The comprehensive search strategy for PubMed is presented in Table 1. The retrieval of other electronic databases is similar to pubmed, which adopts the combination of subject words and key words.

**Table 1:**
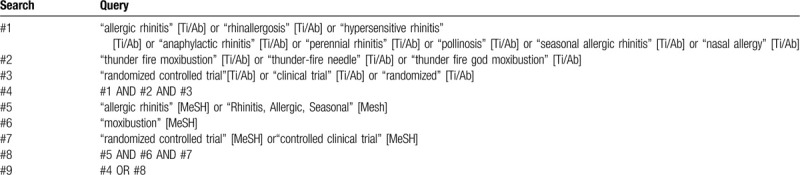
PubMed: will be searched on: July, 2020.

### 2.5 Selection process

Qualified literature will be included according to Cochrane Collaborative System Evaluator's Handbook 5.2.0.^[25]^ Firstly, the literature will be obtained through comprehensive retrieval of the specific databases and imported into the document management software NoteExpress3.0, the repeated documents will be screened out from each database. Secondly, we will read the titles and abstracts preliminarily excluding the literature irrelevant to this study. Thirdly, the selected related clinical studies will be downloaded, and the full text will be screened. Finally, the number of included RCTs will be determined by screening the literature of included studies according to inclusion and exclusion criteria.

**Figure 1:**
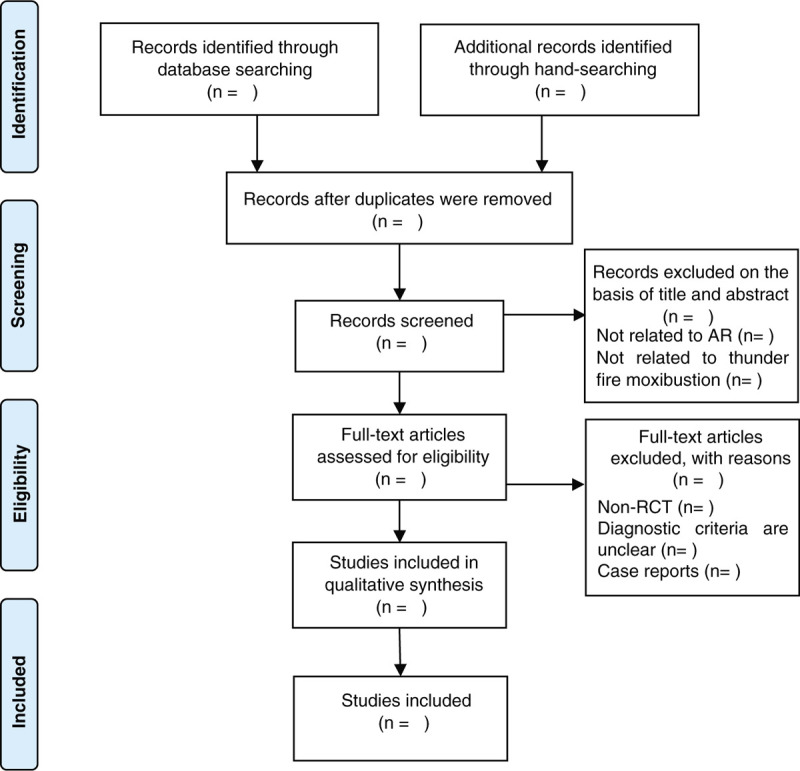
Flowchart of literature selection.

Three independent researchers (TY, QT H, and WX) will follow the procedure strictly. If there is any disagreement, it will be resolved by the fourth researcher (JY). The flowchart is presented in Figure 1.

### 2.6 Data extraction and management

According to the principle of PICOS, the standard data extraction table will be established beforehand. Before the formal data extraction, pre-extraction will be conducted twice to ensure the smooth progress of the formal extraction. Data extraction is carried out independently by 3 researchers (TY, QT H, WX) and cross-checked repeatedly. In case of disagreement, a tripartite discussion will be conducted to reach an agreement or a fourth investigator (JY) will assist in the determination. Meanwhile, intention-to-treat (ITT) analysis will be applied to the missing data. We will use Excel 2007 for data extraction. Relevant contents of data extraction include: title, author, publication time, average age, sample size, disease type, course of treatment, intervention measures, control measures, adverse reactions, outcome indicators, etc. When important data in the literature is missing or incomplete, the study author can be contacted by phone or email.

### 2.7 Assessment of the methodological quality

We will evaluate the methodological quality of qualified RCTs using the Cochrane risk assessment tool^[26]^ according to Cochrane Reviewer's Handbook 5.0. It contains 7 items: random sequence generation, allocation concealment, blinding of participants or doctors, blinding of outcome evaluator, incomplete outcome data, selective outcome reporting and other bias. High (H), low (L), and unclear (U) will be used to evaluate the degree of risk of bias in each item. Three reviewers (TY, QT H, and JY) will cross-check the evaluation results of the included study respectively. If necessary, the corresponding author will be contacted to clarify the issues. In case of disagreement, a tripartite discussion will be conducted to reach an agreement or a fourth reviewer (JX) will assist in the determination.

### 2.8 Level of evidence

The quality of evidence will be assessed by Grading of Recommendations, Assessment, Development and Evaluation (GRADE) system by using the GRADEprofiler 3.6 software. RCTs start with high level of evidence. The level of evidence will be divided into four levels: high, moderate, low and very low. We will down 1 or 2 levels when there are limitations on risk of bias, inconsistency, imprecision, indirectness, and publication bias.^[27]^

### 2.9 Data synthesis

#### 2.9.1 Measures of treatment effect

Meta analysis will be performed using RevMan5.3.0 software. The data are summarized using risk ratios with 95% confidence intervals for binary outcomes or mean difference with 95% confidence intervals for continuous outcomes. If different measurement scales are used, standardized mean difference analyses will be carried out. If the missing data cannot be obtained by contacting the original author, the existing data will be analyzed.

#### 2.9.2 Heterogeneity analysis

Forest plots will be constructed, and the heterogeneity between the qualified studies is tested. Chi-square test and *I*^2^ value are used to test the degree of heterogeneity. When *P* < .1, *I*^*2*^ > 50%, no heterogeneity is considered among the trials, and the fixed effect model will be used for statistical analysis; otherwise, the random effect model will be used. When the clinical heterogeneity between the two studies is large, only descriptive analysis will be performed.

#### 2.9.3 Publication bias

The potential publication bias will be tested using an inverted funnel chart developed by Egger when the number of qualified RCTs are more than 10.^[28]^

#### 2.9.4 Subgroup analysis

If there are enough data, we plan to conduct a subgroup analysis for different groups, including different comparators, treatment times, sorts of western medicine, quality of evidence, and outcome measures.

#### 2.9.5 Sensitivity analysis

The goal of sensitivity analysis is to identify the sources of heterogeneity and confounding factors. If the trials’ data is sufficient, a sensitivity analysis will be performed by excluding the low-quality or high-weight studies one by one.

## 3 Discussion

Presently, AR is mainly treated with antihistamines and intranasal topical glucocorticoids in the nose.^[29]^ Topical intranasal glucocorticoids are the most commonly used treatment for moderate and severe AR in western medicine.^[30]^ However, the long-term use of glucocorticoids can lead to nasal dryness, nosebleed and other adverse reactions, and the incidence of which can be as high as 20%.^[31]^ Moreover, western medicine treatment is not effective for certain patients with moderate to severe AR.^[32]^ In spite of desensitization treatment could effectively improve the symptoms of AR, it has some disadvantages, such as long course of disease, easy recurrence and large side effects. Hence, scientific researchers should intensify research on non-drug therapy to reduce the clinical symptoms and adverse reactions of AR patients.

The current research reports on the mechanism of TFM can be divided into 3 categories, including biological heat transfer characteristics, electrical characteristics and infrared thermal radiation effects.^[33]^ In terms of the characteristics of the biological heat transfer, TFM is based on the theory of meridians and collaterals of traditional Chinese medicine. Through the sensation of meridians and acupoints, it utilizes the physical factors and pharmaceutical chemical factors generated in the combustion process to achieve warm passage of meridians and regulate body functions.^[34]^ When it comes to the electrical properties, the influence of TFM on the electrical characteristics of the meridians are closely associated with the state of the human body. The nonlinear law of “acupoint-organ correlation” is in consistent with the generation and restriction relation of the five-phase theory. During the process of TFM, infrared radiation will be generated.^[35]^ Far infrared ray is easy to penetrate the skin of human body, and heat is transmitted and diffused, while the near infrared ray generated can penetrate deep tissues through capillary network.^[36]^ Near-infrared radiation provides vital energy for the human cell activities and diseased cells. Furthermore, it is conducive to the generation of stimulated resonance of the biolecular hydrogen bond dipole molecules, so as to correct the disorder of energy metabolism in the pathological state by means of the feedback regulation mechanism.^[37]^ Nowadays, in view of the rare reports on the exact mechanism of TFM for treating AR, it is suggested to intensify the clinical research on TFM for AR in order to better guide the clinical practice of acupuncture and moxibustion.

A majority of the trials described complementary and alternative medicine involving the treatment of acupuncture and moxibustion. However, there are no systematic reviews and meta-analysis of TFM for the treatment of AR. This is the first protocol for a systematic review designed to evaluate the efficiency and safety of TFM for AR patients. Thus, we speculate that the results of this review could provide evidence on the efficiency and safety of TFM in treating AR, which will benefit the patients and practitioners.

Nevertheless, the present study has several limitations.

First, although we collect the abundant literature without any language restriction through a comprehensive searching strategy of 9 different databases, we cannot be sure that all relevant RCTs will be included. Secondly, limited by the retrieval conditions, only the Chinese and English databases will be searched, rendering some language bias. Thirdly, we may have difficulty in retrieving raw data from published sources as the corresponding author might not yield raw data. The magazines or references we chose may also be a major source of potential bias. Finally, during the treatment of TFM, it is difficult to blind doctors and patients, which may also cause bias.

## 4 Conclusion

This is the first protocol for a systematic review designed to assess the efficiency and safety of TFM for AR patients. And this review will included comprehensive assessment of methodological quality and the level of evidence. Thus, we speculate that the findings from this review will be used to inform the reference of guideline recommendations for AR.

## Author contributions

**Conceptualization:** Jun Xiong, Ting Yuan, Qiaotong Huang.

**Data curation:** Ting Yuan, Qiaotong Huang, Xue Wang.

**Formal analysis:** YunFeng Jiang, Kai Liao, LingLing Xu.

**Investigation:** Jun Xiong, Ting Yuan.

**Methodology:** Ting Yuan, Qiaotong Huang, Jun Yang.

**Software:** XiaoHong Zhou, LingLing Xu, Kai Liao.

**Supervision:** Jun Xiong, Qiaotong Huang.

**Writing – original draft:** Jun Xiong, Ting Yuan, Qiaotong Huang, Xue Wang.

**Writing – review & editing:** Jun Yang, YunFeng Jiang, XiaoHong Zhou, Kai Liao, LingLing Xu.
